# The Humoral Theory of Transplantation: Epitope Analysis and the Pathogenicity of HLA Antibodies

**DOI:** 10.1155/2016/5197396

**Published:** 2016-12-14

**Authors:** Edward J. Filippone, John L. Farber

**Affiliations:** ^1^Division of Nephrology, Department of Medicine, Sydney Kimmel School of Medicine, Thomas Jefferson University, Philadelphia, PA, USA; ^2^Department of Pathology, Sydney Kimmel School of Medicine, Thomas Jefferson University, Philadelphia, PA, USA

## Abstract

Central to the humoral theory of transplantation is production of antibodies by the recipient against mismatched HLA antigens in the donor organ. Not all mismatches result in antibody production, however, and not all antibodies are pathogenic. Serologic HLA matching has been the standard for solid organ allocation algorithms in current use. Antibodies do not recognize whole HLA molecules but rather polymorphic residues on the surface, called epitopes, which may be shared by multiple serologic HLA antigens. Data are accumulating that epitope analysis may be a better way to determine organ compatibility as well as the potential immunogenicity of given HLA mismatches. Determination of the pathogenicity of alloantibodies is evolving. Potential features include antibody strength (as assessed by antibody titer or, more commonly and inappropriately, mean fluorescence intensity) and ability to fix complement (*in vitro* by C1q or C3d assay or by IgG subclass analysis). Technical issues with the use of solid phase assays are also of prime importance, such as denaturation of HLA antigens and manufacturing and laboratory variability. Questions and controversies remain, and here we review new relevant data.

## 1. Introduction

Central to the humoral theory of transplantation so closely identified with the pioneering work of Terasaki [1, 2] is the ability of the recipient's immune system to produce antibodies against donor mismatched HLA antigens, as well as other polymorphic systems. HLA matching determines that a given transplant can proceed without fear of hyperacute rejection initially, while at the same time minimizing the chances of acute and/or chronic alloimmune mediated rejections in the longer term. In addition, a prominent concern is that sensitization induced by HLA mismatches may impair the ability to receive future transplants should the initial one fail. However, not all mismatches (MMs) result in sensitization, and not all antibodies preclude transplantation. Furthermore, not all antibodies detectable after transplantation injure a graft, whether persisting from pretransplantation or developing* de novo* [3]. In this review, we address recent data relative to 2 important issues regarding antibodies in solid organ transplantation: the role of epitope analysis in optimizing HLA matching and the assessment of the pathogenicity of HLA antibodies.

## 2. Epitopes in HLA Matching

The determination of the three-dimensional structure of an HLA molecule by Cn3D modelling together with amino acid (AA) sequencing led to the definition of polymorphic AA residues on the surface of the molecule accessible to antibody binding. An antibody does not recognize an entire HLA molecule but rather a 15 to 25 AA segment termed an epitope [4]. Epitopes have an area of 700–900 A^2^ within a radius of about 15 Å that represents the* structural* epitope. The corresponding antibody binding surface (paratope) contains 6 complementarity determining regions (CDR), 3 in the hypervariable region of the light chains and 3 in the hypervariable region of the heavy chains.

At the center of an epitope is a polymorphic region of 1 to several AAs within a 3 Å radius, termed an eplet or alternatively the* functional* epitope. These eplets need not be continuous AAs, but they must lie upon protein folding within the 3 Å radius. The third and most variable CDR of the heavy chain lies in the center of the paratope and recognizes the foreign nature of the mismatched eplet that defines the functional epitope. The other 5 CDRs allow for stabilization of the synapse. Eplets are named by their amino acid sequence number followed by one or more AAs. Many epitopes are defined simply by the functional epitope (eplet) alone, whereas others require pairing of that eplet with one or more additional residues within the structural epitope. These secondary configurations may be superficial on the surface of the molecule, where they interact with another CDR. Other times they are hidden, often in the peptide grove, but in this case they have their effect by altering the configuration of the functional eplet.

Duquesnoy developed the HLAMatchmaker program (http://www.HLAMatchmaker.net/) that predicts epitopes based on surface expression of polymorphic amino acid(s) located within a 3 Å radius. This program has the ability to determine epitope specificities of highly sensitized individuals and, by intra- and interlocus subtraction, to compare eplet mismatches between 2 individuals (donor and recipient) [5]. While initially aimed at identifying polymorphisms in three consecutive amino acids (triplets) of class I alleles, newer versions consider 1 to several polymorphic amino acids within a 3 Å radius, including both continuous and discontinuous residues. Class II epitopes have been described as well [6].

The ability of an epitope to be antigenic has been verified by monoclonal antibody binding to single antigen beads by Professor Terasaki's group. Mouse monoclonal antibodies or human alloantibodies absorbed and then eluted from single antigen cell lines, or other sources of single antigens, were tested with single antigen beads (SAB). AAs common to all reactive beads were determined by comparing AA sequences as listed in the Anthony Nolan website. From 1 to 4 AAs common to all reactive alleles that were exposed on the surface and within area of 750 A^2^ were used to define an epitope [7]. By this method, 110 class I, 83 class II, 7 MHC class I chain related gene A (MICA), and 96 cross-reacting epitopes (found in nontransfused healthy males or cord blood) have been defined [8] and named TerEps in Professor Terasaki's honor.

Duquesnoy and Marrari correlated HLA class I TerEps with HLAMatchmaker defined epitopes and found 90% concordance, although about 10% could not be reconciled [9]. Similarly for class II, 49 of 53 HLA-DR TerEps corresponded to HLAMatchmaker eplets, as did 17 of 18 DQ TerEps [10]. Many more eplets are predicted by HLAMatchmaker that have not yet been verified by antibody binding. The international Registry of Antibody Defined HLA Epitopes developed a website (http://www.epregistry.com.br) to record all possible HLA epitopes, denoting those that have been antibody verified [11, 12]. This registry contains 5 databases, HLA-ABC, HLA-DRB, HLA-DQ, HLA-DP, and MICA. As of July, 2016, 289 HLA-ABC epitopes are listed on the website, with 81 noted to be antibody verified. One hundred forty-three HLA-DRB epitopes have been identified, of which 25 are antibody verified.

In the case of HLA-DQ, the *α* and *β* chains are considered separately in the registry with 60 *β*-chain epitopes (16 antibody verified) and 25 *α*-chain epitopes (3 antibody verified) listed. The registry does not consider epitopes defined by specific *α*/*β* parings but determines epitope specificity solely by the *β*-chain [12]. However, Tambur et al. provide evidence that specific *α*/*β*-chain parings can determine a DQ epitope, not either chain in isolation. For example, using Cn3D software and HLAMatchmaker analysis, 39 of 40 recipients with failed allografts had* de novo* DQ antibodies whose paratope covered both *α* and *β* chains [13].

A given alloantibody may be specific for a particular eplet, such that* every* allele carrying that eplet will react with that antibody. Another alloantibody may also react with that same eplet, but* only* if paired with one or more particular additional residue(s). Alleles containing the eplet without the additional residue(s) will not react. These additional eplets are often identical with those of the recipient. This has led to the “non-self-self” theory of epitope recognition, but these additional eplets can also be nonself, or even locus specific monomorphic residues [9]. The additional epitope is usually on the surface, but it can also be hidden with an effect via conformational alteration of the functional eplet [9]. The annotation of such multiply defined epitopes utilizes a “+” sign between the required eplets.

Over half of defined HLA class I epitopes are restricted to a single antigen (private epitopes), whereas the others are shared by 2 or more antigens (public epitopes) [14]. Such public epitopes result in both intra- and interlocus cross-reactions, often referred to as cross-reactive groups (CREGs) [13]. This explains the development of apparent nondonor specific HLA sensitization following solid organ transplantation, although the epitopes are indeed donor specific (discussed below). Also, individual alleles usually contain multiple epitopes. For example, El-Awar et al. found between 6 and 19 epitopes for each HLA-A, HLA-B, and HLA-C antigen [8]. Similarly, HLA-DR antigens had between 8 and 21 epitopes per antigen and DQB chains between 4 and 8.

To better predict and understand the immunogenicity of HLA molecules, the group from Cambridge assessed the physiochemical properties of polymorphic AAs that define eplets, including hydrophobicity and electrostatic charge disparity [15–18]. The binding of antibody to antigen is initially determined by electrostatic interactions [19] and is stabilized by hydrogen bonding, salt bridges, and van der Waals forces [15, 20, 21]. Kosmoliaptsis et al. assessed immunogenicity by determining the AA sequences of HLA molecules (http://www.ebi.ac.uk./imgt/hla/) and then the number of AA mismatches (AAMs) after interlocus subtraction between 32 highly sensitized patients and alleles of a panel of class I SABs exposed to their sera [15]. Both the presence and magnitude of positive responses were highly correlated with the number of AAMs, confirming their prime importance. Independently, however, hydrophobicity mismatch scores (HMS) and electrostatic mismatch scores (EMS, determined as the sum of the difference in isoelectric points of each mismatched AA) also correlated with reaction frequency. The immunogenicity of class II antigens was also shown to be related to the number of AAMs, although it was not independently related to HMS or EMS by multivariable analysis [22].

Kosmoliaptsis et al. generated atomic resolution 3D structural models of HLA class I molecules and calculated the surface electrostatic potential [17]. Using the public Bw4 and Bw6 epitopes as examples, a remarkable consistency of the surface electrostatic potential was shown among all alleles expressing either epitope that reacted with a given alloantibody. AA substitutions that did not affect surface potential did not affect binding, whereas those that did affect surface potential abrogated binding [17, 23].

Finally, immunogenicity of class I HLA mismatched epitopes may depend on the DR phenotype of the recipient. The ability of recipient CD4 T-cells to respond indirectly to donor HLA class I epitopes depends on the ability of such epitope to be presented in the groove of the recipients HLA class II DR molecules on antigen presenting B-cells. Certain anchor amino acids in the peptide are preferred, and this can be predicted [24]. These epitopes have been designated “predicted indirectly recognizable HLA epitopes, HLA class II presented” (PIRCHE-II) [25]. Hence, not all mismatched eplets can be presented to CD4 cells which would inhibit appropriate class switching (IgM to IgG) of B-cells potentially reactive to those eplets. Otten et al. found 49 HLA class I mismatches containing HLAMatchmaker predicted immunogenic eplets among 21 recipients following allograft nephrectomy [25]. DSA were detected for 38 of these mismatches, and these immunogenic epitopes contained a larger number of PIRCHE-II compared to nonimmunogenic ones. Interestingly, 68% of PIRCHE-II epitopes were not part of HLAMatchmaker designated eplets.

## 3. HLA Matching and Epitope Analysis

Current solid organ allocation in the US and elsewhere relies on low resolution serologic HLA matching to determine calculated PRA values. A major question is whether it is worth the time and expense to incorporate epitope analysis in order to better define the immunogenicity of an HLA mismatch, whether by simply determining the AAM load, the EMS, documented antibody binding (TerEps), PRICHE-II epitopes, or the number of epitope mismatches (MMs) using HLAMatchmaker. Data are accumulating in support of these methods as a means of predicting subsequent sensitization, as well as transplant outcomes.

Using the original HLAMatchmaker algorithm that considered defined epitopes based on polymorphic amino acid triplets [26], studies on the utility of epitope matching of class I antigens (HLA-A and HLA-B) in predicting graft survival compared to standard HLA matching have given conflicting results. Some studies show a benefit on graft survival [27, 28], whereas one large study did not [29]. In an earlier study, Laux et al. compared epitope mismatches (EpMMs) at the DPB1 locus (defined as amino acid differences in 4 of the 6 hypervariable regions) with conventional allelic matching in 1,478 kidney retransplant patients [30]. Patients with 2 allelic mismatches but with only 0–2 EpMMs had better 2-year graft survival than those with 1 allelic mismatch but more than 3 EpMMs.

More recently, Wiebe et al. evaluated the development of* de novo* class II (DR and DQ) donor specific antibodies (DSA) in 286 recipients using the current HLAMatchmaker program integrated with antibody verification (TerEps) [31]. Locus specific EpMMs were significantly more frequent in those who developed* de novo* DSA (dnDSA), whereas high resolution typing alone was not helpful. The optimal thresholds below where dnDSA were highly unlikely were 10 epitope mismatches for HLA-DR and 17 for HLA-DQ. Overall, 6 epitopes (3 DFR and 3 DQ) were immunodominant, 5 of which correlated with TerEps. Epitope mismatches at the DP locus were not associated with development of anti-DP DSA.

In a follow-up study of 195 recipients with prospective evaluation of medication adherence, Wiebe et al. evaluated the interaction of noncompliance with these thresholds in predicting late acute rejections and graft loss [32]. The combination of ≥17 DQ EpMMs and medication noncompliance was associated with 3 times the rate of rejection and graft loss as compared to all other groups and ≥10 DR EpMMs plus noncompliance nearly double so. This study suggests that patients under consideration for immunosuppression minimization/withdrawal should have epitope analysis performed.

The Clinical Trials in Transplantation-09 was a multicenter trial aimed at determining if tacrolimus could be safely withdrawn at 6 months from immunologically quiescent patients [33]. It was terminated very early after only 21 patients were enrolled (14 in tacrolimus withdrawal arm) due to safety concerns, as 8 of the 14 withdrawal patients developed acute rejection and/or dnDSA. Using the DQ EpMM cut-off of 17 noted above, 7 of 13 patients with ≥17 DQ EpMMs as defined by HLAMatchmaker developed dnDSA as compared to 0 of 8 with <17 EpMMs (*p* = 0.028). Obviously, these numbers are too small to draw any firm conclusions, but together with the data of Wiebe et al. cited above they do indicate further research is warranted.

Sapir-Pichhadze et al. compared HLA-DRB1/3/4/5, DQA1, and DQB1 EpMM loads using HLAMatchmaker in 52 patients with transplant glomerulopathy (TG) and 104 case-controls [34]. A significantly increased odds ratio (OR) for having TG was found for the highest and middle tertiles of HLA-DR + DQ MMs as compared to the lowest tertile. This held whether modelled as a binary variable or a continuous variable. Surprisingly, DR eplet load appeared to confer a greater risk than DQ eplet load, similar to the findings of Kosmoliaptsis et al. for the immunogenicity of class II HLA [16].

The significance of epitope matching on sensitization following allograft failure has been studied. Singh et al. from our institution studied 66 previously nonsensitized patients that received a kidney transplant that subsequently failed and found that 34 became highly sensitized (cPRA ≥ 80%) [35]. Epitopes were assessed by HLAMatchmaker. Multivariable analysis revealed that DQB1 EpMMs, immunosuppression withdrawal, and the graft intolerance syndrome were significantly associated with high sensitization, whereas HLA-A, HLA-B, DRB1/3/4/5, and DQA1 EpMMs were not. These data highlight the potential importance of epitope matching in those who may require retransplantation, such as pediatric or young adult cases.

Lachmann et al. studied 54 patients with failed kidney allografts: 28 with concurrent nephrectomy/immunosuppression withdrawal (IWD), 14 with just nephrectomy (prior IWD), and 12 with just IWD [36]. HLA antibodies were detectable in 100%, 100%, and 92%, respectively. An increase in breath and intensity of antibodies against class I antigens followed nephrectomy, whereas an increase in class II followed IWD. In a subgroup of 9 patients with concurrent nephrectomy/IWD, epitope specificities were determined by monoclonal antibody binding (TerEps). Overall, they identified 26 mismatched donor epitopes in these 9 patients. Although only 18 of 243 class I antibodies in these patients' serum could be called DSA by traditional matching, 145 of the remaining 225 non-DSA HLA were actually donor epitope specific antibodies (DESA). These data again suggest that epitope matching may reduce sensitization after allograft failure, an issue of prime importance for those requiring retransplantation.

Kosmoliaptsis et al. studied 131 patients with a failed first kidney transplant. Initially, they determined that standard matching (0, 1, or 2 MMs) at HLA-A, HLA-B, HLA-C, DRB1, 3, 4, 5, and DQB1 loci contributed independently to HLA sensitization with an incremental effect [37]. Subsequently, they determined in this same cohort that the AAM score (AMMS), the epitope mismatch score (EpMMS), and the electrostatic mismatch score (EMS) contributed independently to sensitization [18]. All 3 scores independently correlated to HLA-DRB1/3/4/5 and DQ DSA, but only EMS did so with HLA-A and HLA-B DSA. For these analyses they used the algorithms freely available online (http://www.hlaimmunogenicity.org/download/Cambridge_HLA_Class_I_Immunogenicity_Algorithm.xls for class I and http://www.hlaimmunogenicity.org/download/Cambridge_HLA_Class_II_Immunogenicity_Algorithm.xls for class II).

In summary, it is still unclear whether epitope matching should be incorporated into the HLA matching algorithm of all potential kidney transplants. Optimally, it requires high resolution (4 digit) typing, as compared to the low resolution (2 digit) typing currently required by UNOS, which may add significantly to cost and labor [38]. Technology is improving, however, with turnaround time down to several hours. Furthermore, the most likely 4-digit allele can be statistically predicted from serologic typing based on demographic features and haplotype frequencies [39], as is used by the Be The Match Registry of the National Marrow Donor Program (https://bioinformatics.bethematchclinical.org/). Some feel this approach is warranted [38], whereas others feel sufficient information can be obtained from serologic matching with consideration of known CREGs in place of epitopes [40]. Directionality was noted in the protective effect of CREG matching, a finding suggesting that more than just AMMs are involved [40]. The optimal method of epitope analysis is also unclear, be it a simple AMMS, an HLAMatchmaker defined EpMMS, an antibody defined EpMMS, PIRCHE-II, or an EMS.

In our opinion, all highly sensitized patients on the waiting list should have epitope analysis by some method to better define acceptable and unacceptable mismatches. The Eurotransplant Acceptable Mismatch program now incorporates the HLAMatchmaker program for such analysis [41]. In patients likely to need another transplant, epitope analysis for optimal matching of the first transplant is also warranted. At least one pediatric transplant center now incorporates class II epitope matching using HLAMatchmaker when confronted with 2 potential donors (e.g., parents) [42], and we feel this should be the norm. Finally, the degree of EpMM should potentially be assessed in any patient considered for immunosuppression minimization or withdrawal, although more data are clearly required.

## 4. Pathogenicity of HLA Antibodies

Not all antibodies capable of reacting with a kidney allograft are necessarily pathogenic, and significant variability exists in those that are. Initially, the detection of HLA antibodies was dependent on their ability to lyse lymphocytes in the presence of complement, the complement dependent cytotoxicity crossmatch (CDCXM) assay, which is still in use as the final determinant of the safety of a particular transplant [43]. Subsequently, flow cytometry crossmatch (FCXM) technology was developed with higher sensitivity, and it remains in use for the majority of current kidney transplants. In general, outside of desensitization protocols, the presence of DSA detected by either type of crossmatch would abrogate the transplantation, but various exceptions exist [44].

The current standard, however, for evaluating HLA antibodies in most laboratories involves HLA molecules attached to a solid phase matrix, typically a bead, which may contain multiple HLA antigens from multiple cell lines per bead (mixed antigen beads), multiple HLA antigens from a single cell line per bead (phenotypic beads), or a single HLA antigen (single antigen beads or SAB) [45]. The breath of sensitization (“panel reactive antibody” or PRA) is determined along with donor specificity (DSA). These bead-based assays are typically used in conjunction with the cell-based assays (CDCXM, FCXM) to determine if a given transplant should proceed, a decision that also depends on the immunologic risk of a recipient as determined by sensitizing events (prior transplantation, pregnancy, and transfusions), PRA, quality of life on dialysis, life expectancy, and so forth. Various algorithms have been in use [46]. In our laboratory at Thomas Jefferson University Hospital, SABs are used for screening, whereas other centers prefer a screening assay with beads derived from cell lines [47]. In our laboratory all patients undergo autocrossmatch, T and B-cell CDCXM, and FCXM in all live donors and any deceased donor when recipient has any past or current sensitization. If SABs used for screening are completely negative in a patient with no sensitizing events, crossmatching may be superfluous [44, 48].

Features that are possibly significant in determining the pathogenicity of any given DSA include antibody isotype (IgM versus IgG), class specificity (HLA class I versus class II), mean fluorescence intensity (MFI), antigenic specificity (i.e., native HLA class I molecules (nHLA) complexed with *β*2-microglobulin and peptide versus denatured HLA (dHLA) containing *α*-chain only), titer, and ability to fix complement. The latter may be demonstrable by detection of C4d in allograft tissue histologically, or by* in vitro* detection of C1q, C3d, or C4d on synthetic surfaces containing alloantigen epitopes after exposure to patient serum. Also, immunoglobulin subclass determination may be relevant given the differential ability of IgG subclasses to fix complement and recruit inflammatory cells.

Before transplantation, the first order is to predict which DSA will result in a positive crossmatch to obviate unnecessary incompatible offers, as well as to enhance the chances of highly sensitized patients receiving a kidney. In addition, the ability to predict which pretransplant DSA will result in acute antibody-mediated rejection (AAMR) and/or affect long term graft survival (GS) is critical to guide posttransplantation surveillance and immunosuppression. Any DSA detected after transplantation, whether persisting from pretransplantation or developing* de novo*, requires the same scrutiny owing to their documented association with both acute and chronic AMR, as well as graft and patient survival.

## 5. Antibody Isotype

IgM autoantibodies are not pathogenic, although they may result in a positive CDCXM that would be determined by a concurrent positive auto-CDCXM. IgM alloantibodies have been considered to be of low or no potential pathogenicity [49–51], and their presence has even been linked to a more favorable outcome [52]. By contrast, reduced allograft survival has been reported [53]. More recently, a potentially pathogenic role for IgM alloantibodies was detected in eculizumab-treated patients receiving a positive crossmatch kidney transplant, as posttransplantation IgM DSA were found in 3 of 3 such patients with early AAMR, as compared to 1 of 23 without rejection (*p* = 0.006) [54, 55]. In a study of 179 nonsensitized kidney transplant patients, 100 developed DSA, including 53 with IgM alone and 42 with concurrent IgG DSA [56]. A complete IgG subclass profile was not performed, although IgG3 specifically was determined. Only, 5 had IgG DSA without any detectable IgM. IgM alone was not associated with reduced allograft survival, but the 19 IgG3 positive patients with persisting IgM DSA did have reduced allograft survival (*p* = 0.02). IgM alloantibodies may be pathogenic, but more research is obviously needed to clarify this issue. In any case of AAMR not explainable by IgG alloantibodies, we would search for IgM antibodies.

## 6. Class Specificity

Preformed class I antibodies are the major mediators of hyperacute rejection as well as early AAMR. Class II antibodies can also be involved, alone or in combination with class I antibodies [57, 58]. Class specificity of pretransplant DSA does not seem to be an independent variable for determining adverse outcomes by multivariable analysis [59–61]. Class II DSA after transplantation, however, are more deleterious than those against class I for predicting risk of TG and GS [62–64]. This is especially true for DQ antibodies, which are also the most common to develop* de novo* after transplantation [62, 64, 65]. Several factors may explain the prominence of DQ antibodies after transplantation, including linkage disequilibrium with DR [66],* cis/trans* combinations, greater potential peptide variability, and a high degree of self-chain paired with nonself epitope [67]. Further support of the importance of class II loci, especially DQ, comes from a recent registry study from Australia and New Zealand, where mismatches at the DQ locus were significantly associated by multivariable analysis with any rejection or specifically late rejections. In those with 1 or 2 mismatches at the DR locus, any mismatch at the DQ locus was significantly associated with AMR as well [68].

## 7. Mean Florescence Intensity

The single antigen bead assays currently in use are approved as semiquantitative analyses only. The way to assess the potential concentration of antibodies is usually by mean fluorescence intensity (MFI). Only an imperfect correlation exists, however, between MFI and true antibody concentration. Variability in MFI may arise from manufacturing issues regarding density and quality of HLA antigens attached to SAB [69], as well as operator implementation of protocols [45]. Even under conditions of optimal standardization and normalization, the coefficient of variation (CV) between laboratories is 20% to 25% at best [45]. This is above the standard FDA requirement of 15–20% maximal CV to be considered a quantitative test. Numerous factors additionally may alter the relationship between observed MFI and actual antibody concentration and pathogenicity. In states of marked antibody excess where beads are completely saturated, MFI would underestimate antibody concentration. This can be shown by simple dilution, which would not result in a corresponding reduction of MFI. A given antigen may have multiple alleles, each represented on individual beads, and this would tend to lower the MFI relative to an antigen with fewer alleles represented [70]. An antibody directed against a public epitope (e.g., Bw4) would be spread out over multiple beads, resulting in a falsely low MFI, as would relatively low antigen expression with use of phenotype or mixed antigen screening beads [71].

A well-known phenomenon resulting in a marked underestimation of true antibody concentration is often termed the “prozone effect.” Initially, this was ascribed to the inability of the detection antibody to bind the HLA antibody on the bead as a result of interference from bound C1. More recently, the downstream complement activation products C4d and especially C3d have been shown to be responsible [72]. Again, a prozone effect can be uncovered by simple dilution, where the MFI would in this case increase. Also, it can be reversed by disruption of the C1 component of complement by ethylenediaminetetraacetic acid (EDTA), dithiothreitol (DTT), or heating [73]. Heat and DTT denature C1q, and EDTA chelates calcium to inactivate C1q binding [74]. Interference from IgM antibodies has also been reported, [75] as well as from undefined serum factors, and these effects can be mitigated by hypotonic dialysis or DTT [76]. At a minimum, we feel that some methods, such as EDTA or a simple one-time dilution, should be routinely used to eliminate concerns over the prozone effect.

Alloantibodies reacting only with denatured class I antigens would inappropriately inflate the MFI relative to pathogenicity and are discussed below. Also, alloantibodies that do not activate complement* in vitro* may be less pathogenic. This too is discussed below.

Evolution of MFI over time may be more meaningful than an isolated reading. Burns et al. studied the early posttransplant course of 41 crossmatch-positive patients using B-cell flow crossmatch intensity and its correlate, total DSA MFI [58]. All patients had a decrease in intensity by day 4, presumably by adsorption to the graft. These levels remained low in those who did not develop an AAMR but increased significantly in those who did, a result suggesting that serial MFI monitoring may better identify the potential pathogenicity of preformed DSA. In studying patients receiving a FCXM-positive kidney transplant, Kimball et al. showed by SAB analysis that persistence of preformed DSA as compared to their elimination during the course of the first posttransplantation year was significantly associated with AAMR (43% versus 3%), chronic rejection (43% versus 0%), and graft loss (33% versus 5%) [77]. Dieplinger et al. studied 24 patients with dnDSA in the first 2 years after transplantation and found that, over an additional 24 months, 16 lost significant estimated GFR (>25%) [78]. Initial MFI was not different as compared to the 8 with preserved GFR, but the subsequent peak MFI and delta MFI (>20% or >50%) were significantly higher. Altogether, these 3 studies suggest that MFI evolution over time is much more meaningful than any single isolated reading. Nevertheless, more data are clearly needed before immunosuppression can be routinely altered by such information.

Most patients with DSA have antibodies reacting with more than one antigen. In those with multiple DSA, the best way to determine the actual risk for a particular patient remains uncertain. For example, risk may be determined by the single highest MFI. By contrast, the algebraic sum of the MFIs of all DSA may be more relevant. When multiple DSA are present, it would seem reasonable to consider the sum of their MFIs when they share an epitope or epitopes, as opposed to the single immunodominant MFI [47]. Some studies suggest that summing DSA is superior to using the immunodominant MFI when multiple DSA exist [69, 79]. Most recently, Visentin et al. found a greater predictability of positive crossmatches using immunodominant MFI of class I DSA as compared to the sum of class I DSA MFI [80]. The specific use of epitope analysis to answer this question has not been addressed.

## 8. *In Vitro* Complement Activation Assays

The C1q* in vitro* assay detects C1q on SAB after adding human C1q, and many studies have evaluated its role in predicting outcomes. This assay would appear to be an excellent way to assess antibody pathogenicity given the known role of complement in mediating antibody injury [81]. Antibodies may induce injury by mechanisms other than activating complement, however, including antibody-dependent cellular cytotoxicity [82] and direct antibody activation of endothelial cells [83]. Furthermore, C1q-positivity may merely be a surrogate for antibody titer [84, 85]. In order to activate C1q, IgG molecules must be in close proximity to typically form a hexamer [86], which requires a high concentration of antibody. Yell et al. demonstrated that concentration of relatively lower MFI C1q-negative DSA to higher concentration uniformly converted them to C1q-positive, and dilution of C1q-positive DSA converted them to C1q-negative [87]. Low MFI sera may contain C1q-positive DSA, and this is explainable by the prozone effect, at least in some instances [88]. Likewise, high MFI DSA may be C1q-negative [89], possibly if restricted to noncomplement fixing IgG2/IgG4 subclasses (rare), IgM antibodies, abnormalities of IgG Fc glycosylation [90], or due to variable percentages of denatured HLA molecules on individual SAB [91].

In an elegant technical study, Taylor et al. studied 25 highly sensitized, wait-listed patients and looked at the relationship between pan-IgG SAB MFI and C1q-positive MFI in order to assess factors affecting this relationship [91]. Sera were compared neat and following EDTA to abrogate complement interference and simple 1 : 20 dilution to detect very high titers. Also, the percent of denatured HLA class I chains (without *β*-2 microglobulin) was assessed as a contributing factor. There was a poor correlation between neat pan-IgG MFI and C1q-positive MFI (*r* ^2^ = 0.42). The correlation increased following EDTA (*r* ^2^ = 0.57) and dilution (*r* ^2^ = 0.77). A consistent expression of intact HLA (with *β*-2 microglobulin) molecules per SAB was found for all HLA-A and B specificities and most C specificities. Marked variability, however, existed in the amount of denatured HLA molecules between bead populations, ranging from 19% to 91% (mean 69%). The greater the percentage of denatured HLA the lesser the correlation between pan-IgG MFI and C1q-positive MFI, irrespective of which assay was used. Restricting diluted serum to bead populations with ≤30% denatured HLA increased the *r* ^2^ to 0.86. These data suggest that C1q positivity merely reflects MFI if appropriately assessed and does not add significant additional information to justify the excess cost.

Nevertheless, many studies have assessed the ability of C1q-positive DSA to predict adverse outcomes. As we [92] and others [93] have reviewed, at best only conflicting evidence exists to support the pretransplantation use of this assay for such prediction. After transplantation, however, there appears to be a significant relationship [94], and new studies have appeared in the past 2 years to further this notion.

Calp-Inal et al. studied 284 patients with no pretransplant DSA (group 1) and compared them to 405 patients from an earlier era (group 2) with unknown pretransplant status [95]. Over 2.5 years of prospective follow-up, 11% of group 1 patients developed* de novo* DSA, of which 4% were C1q-positive and 7% C1q-negative. AAMR was significantly higher (45%) with C1q-positive DSA as compared to C1q-negative DSA (5%) and no DSA (1%) with *p* < 0.001. The incidence of chronic AMR/TG was significantly higher as well (36% versus 5% versus 2%, respectively, *p* < 0.001). Overall, GS was nonsignificantly reduced with C1q-positive DSA. Similar results were obtained with group 2 patients, with 19% having detectable DSA after transplantation (8% C1q-positive and 11% C1q-negative).

Guidicelli et al. studied 346 nonsensitized patients with DSA evaluation at 2 and 5 years with 10-year follow-up [88]. At 2 years, 25 had* de novo* DSA, 12 C1q-positive and 13 C1q-negative. At 5 years, 30 were* de novo* DSA positive, 8 C1q-positive and 22 C1q-negative. Patients with C1q-negative DSA at 2 years had the same death censored GS (DCGS) at 5 years as DSA-negative patients, but C1q-positive patients had significantly worse DCGS, a result indicating a rather rapid effect on GS if C1q-positive. Those with C1q-positive results at either 2 or 5 years had worse DCGS at 10 years than those without DSA. Interestingly, those with C1q-negative DSA at* both* 2 and 5 years also had worse DCGS than those without DSA, a result suggesting a slower but still pathogenic effect of these C1q-negative DSA.

Lefaucheur et al. studied 125 patients (of 635 consecutive kidney transplants) with DSA detected in the first posttransplant year, with 42% of immunodominant DSA being C1q-positive [96]. By multivariable analysis (MVA) C1q-positivity was independently associated with allograft loss (HR3.6, *p* = 0.03). In a larger follow-up study of 851 consecutive kidney transplants that included the same patients, Viglietti et al. found that 13% were DSA positive at transplantation and 23% were positive after transplantation [97]. C1q-positivity at either time improved significantly the *c* statistic for allograft loss independent of MFI and significantly improved the net reclassification index (NRI) at both time points as well.

In 69 patients with AAMR, Sicard et al. compared the ability of the DSA to activate complement* in vitro* by detecting C1q or C3d with flow bead assays [98]. By MVA, C3d-positivity was significantly associated with graft loss, whereas C1q-positivity was not. This result was validated in an independent cohort. Even C3d+ patients with a low MFI had reduced GS. Similarly, Comoli et al. studied 114 nonsensitized, pediatric, first kidney transplant recipients and found that 39 developed dnDSA at a median of 25 months [99]. Of these, 25 were C1q-positive, and 9 were additionally C3d-positive. Some that were C1q-negative on initial detection progressed to C1q-positive over time, and some of these further progressed to C3d-positive, all with the same antigenic specificity. Any such progression was associated with significant increase in MFI. C3d-positivity significantly enhanced the predictability of dnDSA for 10-year graft survival.

To summarize, while these new data are promising, they must be validated in other populations and tested prospectively. It remains unclear whether determination of* in vitro* complement activating capability by C1q or C3d binding on SABs justifies the additional cost for routine use, although there certainly may be a role in higher risk candidates. Prospective outcome studies with therapy based on such testing could determine the optimal approach.

## 9. IgG Subclass Determination

Given the pathogenic potential of complement activation by IgG HLA antibodies, interest has arisen regarding IgG subclass determination as a means of assessing the pathogenic potential of DSA. The germline order of IgG subclasses begins with IgG3 and proceeds through IgG1, IgG2, and IgG4, sequentially. The strength of complement activation parallels this order with IgG3 and IgG1 considered strong activators and IgG2 and IgG4 weak or not at all. The vast majority of HLA antibodies as detected by SABs are composed of strong complement activators (IgG1 and/or IgG3) alone or in combination with weak/nonactivators (IgG2 and/or IgG4). Isolated weak and/or nonactivators in the absence of strong activators are distinctly unusual, ranging from about 1% [100, 101] to 5% [102, 103] of SAB reactions. Hence, strong activators are nearly always detectable, and the absence of C1q positivity does not rule out their presence [103]. As we reviewed previously [92], the majority of evidence at that time did not support routine subclass determination.

The recent study by Lefaucheur et al. (noted above for C1q) evaluated the characteristics of DSA detected in the first year after transplantation in 125 patients with biopsy, either for indication or as a 1-year protocol, and with 5-year follow-up [96]. These DSA were evaluated for MFI, C1q binding, and subclass distribution. Altogether, 40% of patients had a clinical AAMR, 29% had subclinical AAMR, and 30% were free of AAMR. As in prior studies, IgG1 was most often found (75%), followed by IgG2 (44%), IgG3 (28%), and IgG4 (26%), and 17% had no subclass detectable with a relatively low MFI by pan-IgG. Only 4% had just noncomplement fixing IgG2 and/or IgG4. Overall, 32 of 35 IgG3-positive patients had clinical AAMR, and IgG3 was the only class significantly associated with shortened GS including by MVA. IgG4-positivity was associated with subclinical AAMR and more chronic lesions on biopsy. Viglietti et al. expanded this database to 851 patients and found by MVA that detection of IgG3-positive DSA, either at the time of transplantation or after transplantation, significantly increased the *c* statistic for allograft survival at both time points and improved the NRI as well [97]. While very provocative, these data require independent validation.

## 10. Antibody Titration

Antibody strength is a measure of antibody affinity and avidity and is reflected in the kinetics of antigen-antibody dissociation [104]. Affinity measures the strength of interaction between an antibody paratope and the corresponding epitope and denotes the relative amount of antigen-antibody complex at equilibrium. Avidity is a more global measure of strength and includes affinity, but also valency and structural orientation (e.g., antigen bound to a solid phase matrix). Strength can be determined by serial dilution studies, with the titer being the dilution at which the test becomes negative. Relative strengths of different antibodies can then be defined as the relative MFIs at the highest dilution.

Tambur et al. accumulated over 7000 individual data points from 27 class I assays and 49 class II assays of 55 sensitized patients [104]. A prozone effect, defined here as at least a 100% increase from neat to peak MFI, was detectable for at least one antibody specificity in 71% of patients and for at least one specificity in 40% of class I assays and 65% of class II assays, although overall less than 1% of beads were affected. The effect was often but not always abrogated with EDTA treatment. Looking at the correlation with the ability to fix complement (via the C1q assay), a much higher correlation coefficient was found for the peak MFI on serial dilutions as compared to the standard neat MFI. An even higher correlation was obtained with titers. This study also showed the relative insensitivity of C1q assay, as titers of 1 : 16 to 1 : 32 represented a threshold for C1q positivity for class I antibodies and 1 : 32 to 1 : 64 for class II.

In a follow-up study, the neat IgG MFI, C1q-positive MFI, and antibody titers were compared for their ability to best monitor the effect of desensitization procedures on 40 patients [105]. Titration studies provided a better estimation of strength and more uniformly demonstrated reduction of antibody in response to treatment. Serial titration does add cost, and it remains to be determined by prospective studies the actual role in clinical practice. In our opinion, a one-time dilution may be sufficient to rule out the prozone phenomenon, but serial dilutions would be in order for desensitization protocols or to monitor treatment.

## 11. HLA Antigen Conformation

Class I HLA molecules are present* in vivo* on the cell surface of activated lymphocytes both as intact trimolecular complexes (*α*-chain, *β*2-microglobulin, and peptide) and open conformers (*α*-chain only) [106]. These isolated (“denatured”) *α*-chains (dHLA) may homodimerize and/or bind to intact class I molecules to enhance antigen presentation [107]. They can also heterodimerize with other cell surface receptors, such as the insulin receptor, and alter cell signaling [107, 108]. Such dHLA exist in varying proportions on SAB as a result of different manufacturing techniques as compared to multiantigen screening beads [109], but they may exist on mixed antigen beads as well [110]. Antibodies specific for intact HLA class I can be determined by SABs that lack dHLA, so-called iBeads [111]. Unfortunately, iBeads are no longer commercially available [80]. The relative proportion of intact HLA molecules and dHLA molecules on SABs can be assessed by relative binding of monoclonal antibodies specific for intact HLA (W6/32) or dHLA (HC-10) [112].

Alternatively, antibodies against dHLA can be detected by acid treating regular mixed antigen or SABs, as acid treatment will denature all HLA antigens on the beads. Such treatment would significantly reduce MFI of antibodies reactive with epitopes determined by the trimolecular structure of intact (nondenatured) HLA (nHLA). During the process of denaturing, hidden epitopes may be exposed that are not accessible to binding on intact antigens [14]. Hence, the MFI of a particular serum against a particular bead may markedly increase after acid exposure, thereby indicating the antibodies are directed against dHLA [14, 113]. If the MFI markedly decreases, the antibodies are directed against nHLA. If it stays roughly the same, they are also against nHLA, but anti-dHLA may coexist [113]. Interestingly, antibodies against dHLA may be found in nontransfused healthy male patients as well as in cord blood, a finding suggesting they arise by cross-reactivity with environmental antigens, vaccines, or microorganisms [14, 114].

Other studies indicate anti-dHLA antibodies do not shorten GS. In a study of 156 sensitized patients with 241 class I DSA by regular SAB analysis before transplant, Otten et al. found that 152 DSA were also positive by iBeads and 28 were found only by dHLA beads [112]. Allograft survival was significantly reduced in those with either regular SAB positivity or iBead positivity as compared to nonsensitized patients, whereas in the 20 patients with 28 isolated antibodies to only dHLA, GS was not different from controls. Furthermore, iBead-positive DSA were associated with sensitizing events, whereas dHLA only DSA were not. Likewise, Cai et al. found that 379 (38%) of 994 KT recipients had class I HLA antibodies by mixed antigen bead testing, including 200 with antibodies also reacting with acid-eluted mixed antigen beads (dHLA) and 179 lacking these antibodies (i.e., only containing anti-native HLA antibodies) [110]. Overall GS was equal between sensitized and unsensitized recipients, but it was significantly lower in the 179 that had only anti-native HLA and not in those who also had anti-dHLA.

C1q positivity may signify pathogenicity of antibodies against denatured HLA antigens. Cai et al. studied 975 kidney transplant patients and found that 30% had antibodies against denatured HLA class I, class II, or MICA, using heat-treatment of mixed antigen beads as a means of denaturation [115]. Overall, 8.5% were C1q-positive and 21.5% C1q-negative [115]. Again there was no difference in graft survival comparing those with to those without these antibodies against denatured antigens. However, the 8.5% with C1q-positive dHLA antibodies had lower graft survival compared to recipients with only C1q-negative anti-dHLA or no such antibodies. C1q-positivity was significantly associated with graft failure due to AMR and mixed AMR/CMR.

In summary, MFI on neat serum is clearly an imperfect measure of antibody concentration, strength, and potential pathogenicity. At a minimum, treatment with EDTA or a simple one-time dilution should be routine. Formal titration is indicated in desensitization procedures and possibly in the course of treating acute AMR. The role of* in vitro* complement activating capability as a routine or in the management of the individual patient remains uncertain at this time, as does IgG subclass determination. Consideration of dHLA to explain a high MFI would be most applicable in those without sensitization history. Most importantly, bead-based data must not be used in isolation, but only in conjunction with cell-based assays and with appropriate consideration of sensitization history and other clinical features of any particular patient.
